# Construction of a Nomogram Prediction Model for Mortality Risk Within 14 Days in Patients with Acute Myocardial Infarction and Ventricular Septal Rupture

**DOI:** 10.3390/jcm15082919

**Published:** 2026-04-11

**Authors:** Jie Luo, Ben Huang, Hao-Yu Ruan, Du-Jiang Xie, Gao-Feng Wang, Lei Zhou, Ling Zhou, Shao-Liang Chen

**Affiliations:** 1 Department of Cardiology, Nanjing First Hospital, Nanjing Medical University, Nanjing 210006, China; ljk29184021@163.com (J.L.);; 2 Department of Laboratory Medicine, The First Affiliated Hospital of Nanjing Medical University, Nanjing 210029, China; 3 Department of Cardiology, Shuyang Zhongxing Hospital, Suqian 223600, China

**Keywords:** acute myocardial infarction, ventricular septal rupture, prediction model

## Abstract

**Objective:** This study aimed to develop a nomogram prediction model for predicting 14-day in-hospital mortality in patients with acute myocardial infarction (AMI) and ventricular septal rupture (VSR). **Methods:** Clinical data of 86 hospitalized patients (44 survivors and 42 non-survivors within 14 days) were retrospectively collected in Nanjing First Hospital from 1 March 2015 to 7 August 2025. Lasso regression and multivariable logistic regression were used to identify predictors, which were subsequently incorporated into the nomogram development. The model performance was assessed using area under the receiver operating characteristic curve (AUC), calibration plots, decision curve analysis (DCA), and clinical impact curves, with internal validation via 1000 bootstrap resamples. **Results:** Analysis of lasso regression and multivariable logistic regression analysis identified WBC count (OR = 1.31, 95% CI: 1.01–1.28, *p* = 0.040), D-dimer level (OR = 1.18, 95% CI: 1.01–1.38, *p* = 0.043), early revascularization (OR = 0.22, 95% CI: 0.06–0.88, *p* = 0.032), ventilatory support (OR = 3.48, 95% CI: 1.07–11.29, *p* = 0.038), and infection (OR = 3.97, 95% CI: 1.02–15.42, *p* = 0.047) as independent predictors of 14-day mortality for patients. Based on the results, a prediction nomogram model was constructed. The model achieved an area under the receiver operating characteristic curve (AUC) of 0.866 (95% CI: 0.785–0.946), with sensitivity of 0.857 (95% CI: 0.751–0.963) and specificity of 0.818 (95% CI: 0.704–0.932). Calibration plots demonstrated acceptable agreement between predicted and observed probabilities; decision curve analysis (DCA) and clinical impact curve further confirmed its net benefit and clinical utility. By 1000 bootstrap resampling iterations, the model demonstrated an apparent AUC of 0.864, 95% CI: 0.776–0.938, confirming reasonable discriminative performance. **Conclusions:** In summary, this study developed a clinical interpretable nomogram to estimate short-term (14-day) in-hospital mortality risk in patients with AMI-VSR; it provides a robust and interpretable tool for predicting short-term in-hospital mortality.

## 1. Introduction

Ventricular septal rupture (VSR) is a rare but life-threatening mechanical complication after acute myocardial infarction (AMI). According to reports of The National Inpatient Sample database (2009–2020), the incidence of VSR after AMI is approximately 0.19% [1]. The incidence of VSR has effectively declined from ~2% to ~0.25% with early revascularization strategies [2]. However, in early stages, the mortality rate of VSR is still high, approximately 60% within 24 h in untreated patients and 67–82% within 1 week of conservative medical management; it has a 1-year survival rate of only 7% [3,4].

The implementation of early supportive therapy combined with subsequent surgical procedures has been shown to significantly improve patients’ clinical outcomes, with one-year survival rates now increasing to 60% [5,6]. Studies indicate that patients generally present with relatively stable hemodynamic status and scar tissue surrounding the site of perforation within 2 to 6 weeks following myocardial infarction [7,8]. It is widely accepted that the optimal timing for surgical repair is at least 4 weeks [8], for percutaneous occlusion it is at least 2 weeks [9]. However, the cumulative mortality rate of conservative therapy in 2 weeks is 70% [7,10,11]. Consequently, the prompt identification of patients with a high risk of early mortality risk is of critical importance.

Previous studies have identified several factors potentially predictive of early mortality, including the presence of cardiogenic shock, anterior acute myocardial infarction, delayed coronary reperfusion, and the absence of ventricular aneurysm formation [1,4,10,12,13]. Nevertheless, a validated clinical model specifically designed to predict short-term mortality in patients with VSR remains to be established.

Therefore, careful investigations of VSR risk factors and a comprehensive risk assessment are essential. Given that this complication is rare but carries an extremely high mortality rate, improving early risk stratification is of great significance for clinical decision-making and resource allocation. Our study aimed to identify critical risk factors and establish a predictive model of short-term mortality in patients with VSR.

## 2. Materials and Methods

### 2.1. Population

**Figure 1:**
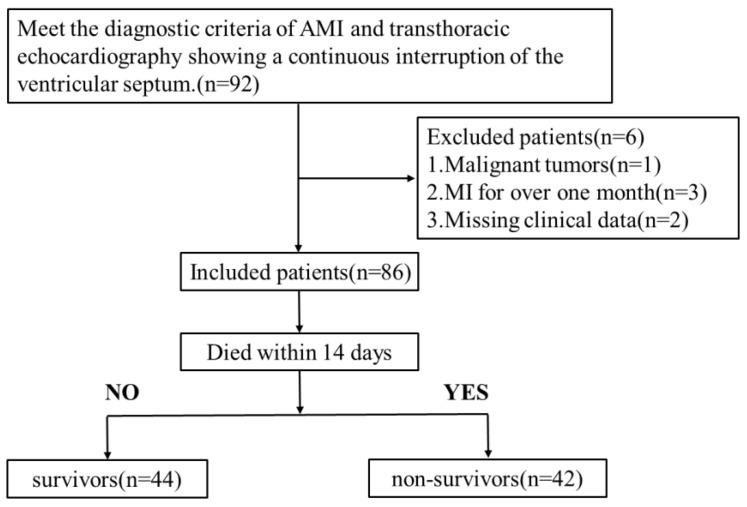
Flowchart for screening the included patients.

In our retrospective study, patients with VSR hospitalized at Nanjing First Hospital from 1 March 2015 to 7 August 2025 were collected. The inclusion criteria included: (1) meet the diagnostic criteria of AMI and (2) transthoracic echocardiography showing a continuous interruption of the ventricular septum. The exclusion criteria were: (1) congenital ventricular septal defects; (2) malignant tumors; (3) autoimmune diseases; (4) MI for over one month; and (5) missing clinical data. Figure 1 showed the flowchart for selecting the included patients. This study was conducted according to the guidelines of the Declaration of Helsinki, and approved by the Ethics Committee of Nanjing First Hospital (protocol code: No.KY20260220-KS-01, date of approval: 2 February 2026).

### 2.2. Data Collection

Clinical variables were extracted from the hospital’s electronic medical record (EMR) system. All laboratory test and imaging examination results were collected within 24 h of the confirmed diagnosis of VSR. It is worth noting that all the variables included in this study were collected before the occurrence of the outcome event. The specific time points for collecting the variables were shown in Supplementary Figure S1. The collected data included: (1) Baseline data: gender, age, height, weight, body mass index (BMI), medical history, baseline (upon admission) vital signs, Killip class, and anterior wall infarction. (2) Laboratory indicators: white blood cells (WBC), hemoglobin (HGB), platelet (PLT), serum K^+^ (mmol/L), Na^+^ (mmol/L), albumin, alanine aminotransferase (ALT), aspartate aminotransferase (AST), creatinine (CREA), urea nitrogen, uric acid, creatine kinase (CK), creatine kinase isoenzymes (CK-MB), troponin I (TNI), N-terminal pro-brain natriuretic peptide (NT-proBNP), D-dimer, triglycerides (TG), total cholesterol (TC), high-density lipoprotein (HDL), low-density lipoprotein (LDL), total bilirubin (TBIL), direct bilirubin (DBIL), and indirect bilirubin (IBIL). (3) Echocardiographic indicators: left ventricular end-diastolic dimension (LVEDD), left ventricular ejection fraction (LVEF), pulmonary artery systolic pressure (PASP), size, and location. (4) Other parameters: AMI to VSR (A to V), AMI to Door (A to D), early revascularization, cardiac shock (CS), intra-aortic balloon pump (use of IABP), use of ventilatory support: non-invasive positive pressure ventilation (NIPPV) or assisted mechanical ventilator (AMV), use of vasoactive drug, and infection. These variables were intentionally retained to reflect a real-world clinical trajectory whose inclusion precludes causal interpretation.

### 2.3. Lasso Regression Analysis

Lasso regression is a widely adopted penalized regression technique in statistics and machine learning. It extends ordinary linear regression by incorporating an L1 penalty term—the sum of the absolute values of the regression coefficients—into the loss function. By tuning the regularization parameter λ, lasso shrinks coefficient estimates toward zero, thereby enabling automatic variable selection and yielding sparse, interpretable models. Its utility is particularly pronounced in high-dimensional settings, where it mitigates overfitting and improves model generalizability, even in the presence of multicollinearity. In this study, lasso regression was implemented using the “glmnet” package in R 4.4.2. The optimal λ value was determined via ten-fold cross-validation using the “cv.glmnet” function.

### 2.4. Logistic Regression Analysis

Subsequently, logistic regression analysis was used to further identify the independent predictors associated with the patients’ mortality within 14 days, the odds ratios (OR) of the variables as well as the 95% confidence intervals were calculated. In this analysis, logistic regression was performed using the “glm” function from the “stats” R package. For the multivariate analysis, variables with a univariate *p* < 0.05 were entered into a bidirectional stepwise regression using the “stepAIC” function from the “MASS” R package.

### 2.5. Construction and Evaluation of the Nomogram Prediction Model

Based on the results of the multivariate logistic regression analysis, the “rms” package in R 4.4.2 software was used to build the nomogram model. Subsequently, the receiver operating characteristic (ROC) curve, calibration curve, decision curve analysis (DCA), and clinical impact curve (CIC) were drawn. The Hosmer–Lemeshow test and the Bootstrap resampling method (repeated sampling 1000 times) were used to evaluate the predictive value, calibration degree, clinical applicability, and fitting degree of the nomogram model.

### 2.6. Statistical Analysis

Statistical analysis was performed using R 4.4.2 (R Foundation for Statistical Computing, Vienna, Austria) and GraphPad Prism 10 software (GraphPad Software, San Diego, CA, USA). The normality of the data was assessed using the Kolmogorov–Smirnov test. For continuous variables following a normal distribution, data are expressed as mean ± standard deviation ($\overset{–}{x}$ ± SD), and the independent samples *t*-test was performed. For non-normally distributed data, results are presented as median with interquartile range [M (Q1, Q3)], and the Wilcoxon rank-sum test was applied. Categorical variables are summarized as frequency (percentage) and analyzed with the chi-square test or Fisher’s exact test. Lasso and logistic regression analysis were performed to identify the risk indicators. The nomogram prediction model was constructed using the rms package in R 4.4.2. The goodness-of-fit of the model was evaluated using the Hosmer–Lemeshow test. The discriminatory performance of the model was assessed by plotting the ROC curve and calculating the area under the curve (AUC), sensitivity, and specificity. To evaluate the clinical utility of the model, DCA and CIC analysis was performed to assess the net benefit across different threshold probabilities. Furthermore, the stability of the model was validated using the Bootstrap method with 1000 resamples, and a calibration curve was plotted to visualize the agreement between predicted and observed outcomes. All statistical tests were two-tailed, and a *p*-value less than 0.05 was considered statistically significant.

## 3. Results

### 3.1. Analysis of Baseline Data Clinical Parameters in VSR Patients

In this study, 86 patients were categorized into a survival group (n *=* 44) and a dead group (n *=* 42) based on their clinical outcomes within 14 days of hospitalization. The baseline clinical characteristics of the two groups were presented in Table 1. Compared with the survival group, the death group exhibited statistically significant differences in the following variables: WBC, CK, CK-MB, TNI, D-dimer, early revascularization, ventilatory support, vasoactive drug, and infection.

### 3.2. Results of Lasso Regression Analysis

**Figure 2:**
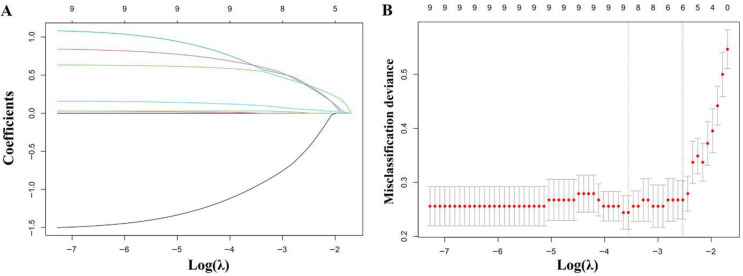
Results of lasso regression analysis. (**A**) The variable selection process via lasso regression analysis, where increasing the regularization parameter λ progressively shrinks variable coefficients toward zero, ultimately excluding irrelevant or weakly associated predictors. The different colored lines in the figure represent different variables. (**B**) The cross-validation curve for lasso regression analysis. The two vertical dashed lines indicate λ.min and λ.1se.

To further identify key predictive variables, the aforementioned significant variables were subjected to lasso regression analysis, and the results were presented in Figure 2. The ten-fold cross-validation method was employed to determine the optimal penalty coefficient lambda (λ). Figure 2A illustrated the variable selection process using lasso regression. The Lasso regression cross-validation curve analysis revealed that when λ reached its value lambda.1se, the prediction error was minimized (Figure 2B). At this optimal λ value, 6 variables were retained: WBC, D-dimer, early revascularization, ventilatory support, vasoactive drug, and infection.

### 3.3. Results of Logistic Regression Analysis

Based on the variables selected through lasso analysis, the logistic regression analysis was performed (Table 2). Multivariable analysis identified WBC count (OR = 1.31, 95% CI: 1.01–1.28, *p* = 0.040), D-dimer level (OR = 1.18, 95% CI: 1.01–1.38, *p* = 0.043), early revascularization (OR = 0.22, 95% CI: 0.06–0.88, *p* = 0.032), ventilatory support (OR = 3.48, 95% CI: 1.07–11.29, *p* = 0.038), and infection (OR = 3.97, 95% CI: 1.02–15.42, *p* = 0.047) as independent predictors of 14-day mortality for patients.

### 3.4. Establishment and Analysis of the Nomogram Prediction Model

**Figure 3:**
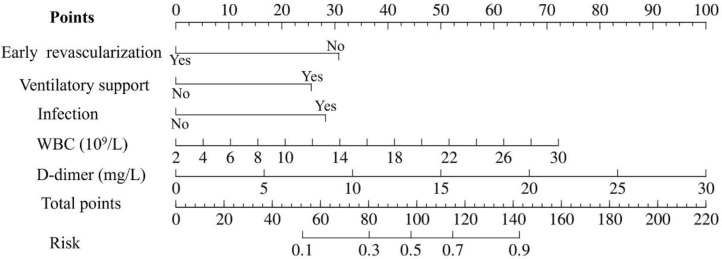
A nomogram prediction model for mortality risk within 14 days in patients with acute myocardial infarction and ventricular septal rupture.

Based on the results of multivariate logistic regression analysis, a prediction nomogram model for the occurrence of death within 14 days in patients was constructed, as shown in Figure 3.

In this model, early revascularization, ventilatory support, infection, WBC count, and D-dimer level were each assigned a point score. The total score was calculated by summing the points for all applicable factors. To estimate 14-day mortality risk, the total score was located on the “Total Points” axis of the nomogram, a vertical line was then drawn from total point to intersect the “Risk” axis, yielding the corresponding probability of death within 14 days.

**Figure 4:**
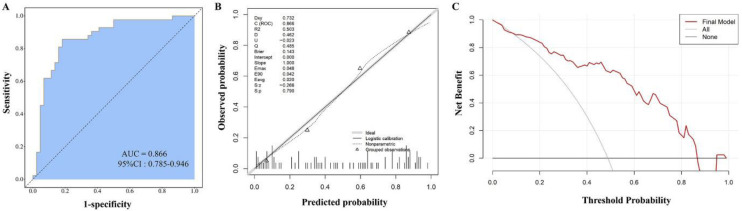
Evaluation and analysis of the nomogram prediction model. (**A**) ROC analysis of the nomogram prediction model, the dashed line represents the diagonal. (**B**) Calibration curve. (**C**) Clinical decision curve analysis.

Subsequently, we further assessed the performance of the model using ROC analysis. The area AUC was 0.866, 95% CI: 0.785–0.946, indicating good discriminative ability (Figure 4A). Moreover, the model demonstrated the sensitivity of 0.857, 95% CI: 0.751–0.963, and a specificity of 0.818 (95% CI: 0.704–0.932). The positive predictive value was 0.818 (95% CI: 0.704–0.932), and the negative predictive value was 0.857 (95% CI: 0.751–0.963). Analysis of the calibration curve indicated that the nomogram model’s predicted probability of death within 14 days for VSR patients showed reasonable agreement with the actual observed probability (Figure 4B). In addition, the DCA results indicated that the use of the nomogram model had good clinical practical value (Figure 4C). These findings suggested that the model possessed good discriminative ability and performance.

To further evaluate the predictive performance of the 5-variable nomogram, we performed a nested model analysis by sequentially adding variables (Table 3). Results showed that the full 5-variable nomogram achieved a higher AUC (0.866), sensitivity (0.857), and specificity (0.818) than the other four models, indicating improved overall predictive performance. In addition, since the AUC values of both Model 4 and Model 5 were greater than 0.8, the overall net reclassification improvement was calculated. The NRI was 0.183, 95% CI: 0.034–0.336, *p* = 0.017, suggesting that the full 5-variable nomogram significantly improved risk classification. Collectively, these nested model comparisons demonstrated that the nomogram provided meaningful incremental value.

### 3.5. Analysis of Internal Validation by the Bootstrap Method

**Figure 5:**
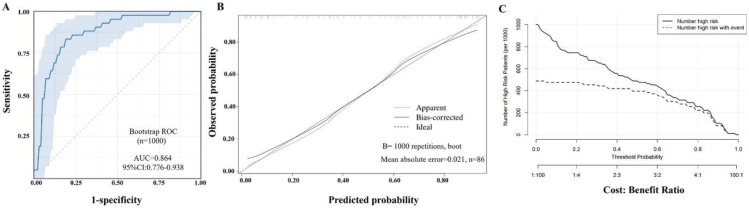
Analysis of internal validation by the bootstrap method. (**A**) ROC analysis of the nomogram prediction model by the bootstrap method (n = 1000); the apparent AUC was 0.864, 95% CI: 0.776–0.938. The light blue shading represents the 95% confidence interval of the ROC curve, and the dotted line at the diagonal is the diagonal line. (**B**) Calibration curve. (**C**) Clinical impact curve.

Following 1000 bootstrap resampling iterations (Figure 5A), the apparent AUC was 0.864 (95% CI: 0.785–0.946). After optimism correction, the optimism-corrected AUC was 0.826 (95% CI: 0.756–0.927), with an estimated optimism of 0.038, indicating reasonable discrimination performance. The bootstrap calibration plot further revealed good agreement between predicted and observed outcomes, with a mean absolute error of 0.021 (Figure 5B). The Hosmer–Lemeshow goodness-of-fit test yielded a χ^2^ value of 12.688 with a corresponding *p*-value of 0.123, suggesting that the model demonstrated a good overall fit, with no statistically significant discrepancy between the predicted probabilities and the observed outcomes. In addition, the clinical impact curve (CIC) was generated to assess the clinical utility of the risk prediction nomogram (Figure 5C). CIC curve quantifies the number of individuals classified as high-risk across varying risk thresholds. The CIC visually indicated that the nomogram had superior overall net benefits, suggesting that the model had significant predictive value. These results collectively suggested that the nomogram model demonstrated reasonable calibration and may be useful for clinical risk prediction.

## 4. Discussion

VSR still remains a critically severe complication after acute myocardial infarction. VSR occurring within 24 h is related to endocardial avulsion (68%), while delayed VSR has a relationship with expansion of transmural necrosis [10,14]. According to data from the database of the Society of Thoracic Surgeons, the 30-day mortality for patients with VSR was 42.9%. Mortality was significantly higher when intervention occurred within 7 days (54.1%) compared to after 7 days (18.4%) [8]. Both interventional closure and surgical repair have been proven to reduce overall mortality in patients with VSR, while a considerable number of patients die in the early stages [15], which identifies those patients who can’t wait for surgery. In our study, we identified 5 critical features for assessing early morality risk of patients with VSR by lasso and logistic regression, including early revascularization, ventilatory support, infection, WBC count, and D-dimer.

Previous research has demonstrated that the interval between the onset of myocardial infarction (MI) and the detection of VSR predominantly occurs within 1 to 7 days [16], with a particular concentration in patients presenting within 24 h of symptom onset who often exhibit extensive transmural infarction [17]. Therefore, the timing of rupture is positively correlated with the severity of the condition. Nevertheless, not all MI patients undergo immediate echocardiographic evaluation upon admission, nor do they receive daily echocardiographic examinations during hospitalization. Consequently, many instances of delayed rupture are identified only after the onset of hemodynamic instability, frequently resulting in postponed therapeutic intervention [13].

Existing studies have indicated that successful coronary revascularization reduces the risk of VSR by approximately 80%, although reperfusion injury may induce hemorrhagic necrosis of the myocardium [4,12,18]. As a result, the majority of ruptures in patients occur within 24 h after reperfusion [19]. Our findings suggest that early revascularization contributes positively to the short-term prognosis of patients with VSR. Previous data has also reported that reperfusion injury can lead to myocardial hemorrhagic necrosis, while the resultant rupture may be smaller in size, it tends to progress rapidly. However, due to the reduced infarction size, short-term survival rates are superior compared to patients who do not receive revascularization therapy [20]. Conversely, patients who do not receive reperfusion treatment are at an elevated risk of developing cardiogenic shock subsequent to rupture, requiring prolonged circulatory support, and exhibiting higher rates of in-hospital mortality [21].

Once a VSR forms, the increasing left-to-right shunt ultimately results in cardiogenic shock. Approximately 50% of patients with VSR developed cardiogenic shock in the CAUTION study [7]. The mortality risk increases to over 90% with the onset of circulatory collapse [2]. Therefore, for these patients, early recognition and prompt intervention are crucial to maintaining circulatory stability and reducing mortality. By increasing cardiac output, reducing the left-to-right shunt, and improving myocardial ischemia, thereby alleviating cardiac afterload, vasoactive drugs serve as a cornerstone in maintaining hemodynamic stability for patients with VSR. Nevertheless, the use of vasoactive drugs has some adverse effects, and previous studies have also demonstrated that medical treatment alone fails to improve clinical outcomes [22]. Another cornerstone of hemodynamic stability is mechanical circulatory support (MCS) [7]. An intra-aortic balloon pump (IABP) is used worldwide in cardiogenic shock secondary to myocardial infarction due to its ease of use and relatively low cost. It primarily maintains circulatory stability by increasing cardiac output and improving coronary perfusion [23,24]. Nevertheless, the routine application of IABP failed to improve survival when compared to medical therapy [25]. We also observed that use of IABP did not lead to a reduction in 14-day mortality risk in patients. Early application of advanced MCS, such as Impella, TandemHeart, and VA-ECMO, may provide a potential benefit to the prognosis of VSR shock [23].

In patients with VSR, pulmonary edema is a prevalent and serious complication that can lead to respiratory failure, thus necessitating respiratory support [23]. Both invasive and non-invasive ventilation have been shown to be pivotal therapeutic pillars that stabilize patients by improving oxygenation, reducing work of breathing and oxygen consumption, and alleviating edema. However, the inclusion of ventilatory support in the model reflects severity of hemodynamic compromise and respiratory failure rather than a direct pathophysiological role of mechanical ventilation in outcomes. As noted in the limitations, this variable is treatment-dependent and should be interpreted as an indicator of early clinical status rather than a causal predictor.

Infection constitutes a significant risk factor for clinical deterioration in patients suffering from VSR [11]. The interplay between infection and VSR contributes to compromised renal perfusion and hepatic congestion thereby more than doubling the associated mortality rate [11]. Infection was identified as a predictor of poor outcomes. However, this association likely reflects the broader clinical context of systemic inflammatory response, sepsis-related hemodynamic compromise, and multi-organ dysfunction, rather than a direct interaction between infection and VSR. Our model does not establish causality, and this finding should be interpreted as a prognostic association within a high-risk population. Leukocytes play a central role in this inflammatory cascade. They are transported via the bloodstream to the microvasculature, where they may impair endothelium-dependent vasodilation and vascular function, predominantly through mechanisms involving oxidative stress [26,27]. The resultant injury to cardiomyocytes and the extracellular matrix collagen elevates the risk of cardiac rupture. Consequently, numerous studies have identified leukocytosis as a critical early prognostic marker for short-term mortality in patients with VSR [6,11,17,28]. Empirical evidence indicates that each increment of 1 × 10^9^/L in white blood cell count corresponds to a 14% to 62% increase in mortality risk, with a pronounced escalation observed when counts exceed 15 × 10^9^/L [29]. The integration of leukocyte counts with additional clinical parameters, such as the Sequential Organ Failure Assessment (SOFA) score and lactate concentrations, enhances the precision of risk stratification in this patient population [29].

D-dimer is a degradation product of cross-linked fibrin, and its elevated level directly reflects activation of the coagulation system and a state of secondary hyperfibrinolysis. The D-dimer level is significantly associated with the severity long-term prognosis in patients with VSR [30]. A high D-dimer level indicates a hypercoagulable state, often suggesting a larger infarct size, more severe cardiac dysfunction, and a higher likelihood of concomitant cardiogenic shock [31]. Second, D-dimer provides important clues for selecting the timing and modality of treatment. Studies have shown that if D-dimer does not show a decreasing trend preoperatively, the risks of postoperative residual shunt, device displacement, or myocardial reinfarction are significantly increased [32]. In patients managed conservatively, persistently high D-dimer levels often suggest that the condition is difficult to stabilize, necessitating more aggressive mechanical circulatory support to gain a window for surgery [17]. Finally, D-dimer is an independent risk factor for prognosis. Clinical data indicate that the D-dimer level at admission is positively correlated with 30-day mortality and long-term cardiac mortality in patients with VSR. Even among those who successfully undergo surgery, patients with extremely high preoperative D-dimer have significantly longer hospital stays and a higher risk of poor prognosis due to postoperative complications such as right ventricular dysfunction, coagulation disorders, and infection compared with those who have only mildly elevated D-dimer [8]. Elevated D-dimer was associated with increased mortality in our model. While this biomarker is biologically plausible in the context of thrombosis and fibrinolysis, it should be interpreted primarily as a prognostic marker reflecting the severity of systemic prothrombotic and inflammatory states commonly seen in patients with cardiogenic shock, rather than as evidence of a direct causal mechanism. Dynamic monitoring of D-dimer changes in clinical practice helps to identify high-risk patients early and optimize treatment strategies.

With respect to the above, this study successfully developed a nomogram model that attained an apparent AUC of 0.866, (95% CI: 0.785–0.946); after optimism correction, the optimism-corrected AUC was 0.826 (95% CI: 0.756–0.927), indicating reasonable performance. To further evaluate the predictive performance of the 5-variable nomogram, we performed a nested model analysis by sequentially adding variables. Results showed that the 5-variable full nomogram provided a more meaningful incremental value than 2–4 variables. Collectively, these improvements enable the model to achieve an optimal balance between predictive accuracy and clinical applicability.

Nonetheless, it is important to acknowledge the limitations of the present study. First, in the final multivariate logistic regression model, five predictor variables were included. Consequently, the effective events-per-variable (EPV) ratio was calculated as: EPV = number of endpoint events/number of predictors in the final model = 42/5 = 8.4. Therefore, the EPV was borderline, given the number of predictors retained in the final model. In the future, the number of the research population will be increased to further validate the performance of the model. Second, similar to the majority of predictive modelling studies, the data utilized in this investigation were obtained from a single center. A notable limitation of this study is the absence of external validation. As the model was trained on data from a single center, it increases a potential risk of overfitting, thereby potentially compromising its generalizability. Consequently, the application of this model to other regions or populations warrants caution. Future studies involving external validation are necessary to adequately assess the model’s performance and broader applicability. In addition, the inclusion of treatment variables as predictors introduces the risk of treatment leakage and may lead to an overestimation of model performance—particularly problematic for a prediction tool intended for early risk stratification. On the other hand, these variables are inherently treatment-dependent and driven by disease severity, they do not represent purely baseline predictors. This limits causal interpretability and constrains the applicability in early decision-making contexts. Moreover, due to the retrospective nature of this study and the fact that data were collected from a real-world clinical setting, several clinically relevant variables were not uniformly available for all patients and therefore could not be included in the final prediction model. Finally, as a retrospective study, it is inherently susceptible to certain degrees of selection and information bias. Consequently, future prospective studies are warranted to validate these findings.

Taken together, the variables retained in our model—including infection, D-dimer, and ventilatory support—are best understood as collective indicators of systemic deterioration, encompassing cardiogenic shock, systemic inflammation, and progressive organ failure. Rather than suggesting discrete mechanistic pathways, our model provides a clinically useful prognostic tool for risk stratification based on early clinical status. Clinicians should interpret these variables within the broader context of overall illness severity rather than as isolated therapeutic targets. We hope this model can serve as an effective clinical tool by aiding in risk assessment, enabling early detection of high-risk patients, guiding personalized therapy, and thereby potentially enhancing prognoses. Regardless, further validation through multi-center, large-scale cohorts is essential to confirm its universal applicability.

## Figures and Tables

**Table 1:** Analysis of clinical parameters in VSR patients.

Variables	Total (n = 86)	Survival Group (n = 44)	Dead Group (n = 42)	Statistic	*p*-Value
Age (years)	72.50 (66.25, 78.00)	72.50 (65.75, 78.00)	72.50 (67.00, 79.25)	Z = −0.19	0.849
Gender, *n* (%)				χ^2^ = 3.07	0.080
Female	47 (54.65)	20 (45.45)	27 (64.29)		
Male	39 (45.35)	24 (54.55)	15 (35.71)		
Temperature (°C)	36.50 (36.30, 36.60)	36.50 (36.30, 36.60)	36.55 (36.32, 36.70)	Z = −1.42	0.155
SBP (mmHg)	110.00 (100.00, 122.75)	110.00 (100.75, 129.00)	110.00 (99.25, 120.75)	Z = −0.64	0.522
DBP (mmHg)	71.50 (65.25, 83.00)	70.00 (65.00, 83.25)	73.00 (66.25, 82.50)	Z = −0.39	0.697
HR (beat/min)	100.00 (90.25, 112.00)	99.00 (88.75, 109.25)	103.00 (93.75, 114.00)	Z = −1.33	0.183
WBC (10^9^/L)	12.00 (8.70, 16.54)	9.62 (7.02, 12.82)	14.96 (10.57, 17.18)	Z = −3.72	<0.001
Hemoglobin (g/L)	119.50 (106.50, 128.75)	118.00 (105.75, 127.25)	121.00 (108.00, 130.75)	Z = −0.64	0.522
Platelet (10^9^/L)	231.50 (145.00, 296.50)	221.50 (120.50, 294.25)	233.50 (150.50, 304.75)	Z = −0.50	0.619
K^+^(mmol/L)	3.94 (3.62, 4.38)	3.92 (3.48, 4.26)	4.06 (3.75, 4.71)	Z = −1.86	0.063
Na^+^ (mmol/L)	136.35 (132.72, 138.28)	137.00 (134.00, 138.60)	135.35 (131.00, 138.17)	Z = −1.82	0.068
Albumin (g/L)	33.10 (30.72, 36.88)	33.35 (31.43, 36.35)	32.90 (30.13, 37.08)	Z = −0.83	0.409
CREA(μmol/L)	102.85 (66.50, 197.85)	84.60 (62.00, 152.75)	131.25 (77.53, 217.62)	Z = −1.89	0.058
CK(U/L)	251.00 (83.25, 790.50)	141.00 (60.00, 434.50)	537.50 (174.00, 973.25)	Z = −2.84	0.004
CK-MB(U/L)	21.00 (13.25, 67.00)	18.50 (11.75, 54.17)	42.00 (17.25, 91.75)	Z = −2.22	0.027
NT-proBNP (pg/mL)	8857.78 (3110.30, 10000.00)	6235.43 (2962.10, 10000.00)	10000.00 (4297.60, 10000.00)	Z = −1.63	0.103
TNI (ng/mL)	3.98 (0.95, 17.56)	1.20 (0.23, 8.79)	7.56 (3.29, 20.89)	Z = −3.54	<0.001
D-dimer (mg/L)	2.05 (1.05, 4.04)	1.56 (0.79, 2.40)	2.63 (1.15, 6.18)	Z = −2.70	0.007
Urea (mmol/L)	12.18 (7.88, 19.89)	11.35 (7.63, 17.09)	12.98 (8.06, 22.84)	Z = −1.27	0.206
Uric acid(µmol/L)	495.00 (366.75, 630.00)	441.00 (361.93, 523.75)	533.50 (396.00, 838.75)	Z = −1.78	0.076
TG (mmol/L)	1.32 (1.01, 1.65)	1.27 (1.00, 1.49)	1.33 (1.05, 1.94)	Z = −1.70	0.089
TC (mmol/L)	3.65 (2.95, 4.59)	3.59 (2.94, 4.05)	3.69 (2.97, 4.64)	Z = −0.65	0.514
HDL (mmol/L)	0.77 (0.63, 0.95)	0.83 (0.64, 0.98)	0.76 (0.61, 0.87)	Z = −1.40	0.160
LDL (mmol/L)	2.17 (1.86, 2.88)	2.14 (1.81, 2.81)	2.23 (1.90, 2.94)	Z = −0.85	0.397
TBIL (µmol/L)	14.80 (9.20, 24.70)	12.45 (8.38, 20.30)	15.75 (11.30, 27.18)	Z = −1.61	0.108
DBIL (µmol/L)	5.25 (3.90, 11.47)	5.20 (3.77, 9.93)	5.95 (4.10, 14.67)	Z = −1.21	0.225
IBIL (µmol/L)	7.45 (4.95, 11.38)	6.95 (5.05, 11.00)	7.70 (5.15, 11.88)	Z = −0.88	0.380
Early revascularization, n (%)				χ^2^ = 5.93	0.015
No	66 (76.74)	29 (65.91)	37 (88.10)		
Yes	20 (23.26)	15 (34.09)	5 (11.90)		
A to D, *n* (%)				χ^2^ = 0.38	0.535
0(≤12 h)	36 (41.86)	17 (38.64)	19 (45.24)		
1(>12 h)	50 (58.14)	27 (61.36)	23 (54.76)		
Anterior wall infarction, n (%)				χ^2^ = 0.01	0.912
No	68 (79.07)	35 (79.55)	33 (78.57)		
Yes	18 (20.93)	9 (20.45)	9 (21.43)		
CS, n (%)				χ^2^ = 2.30	0.130
No	42 (48.84)	25 (56.82)	17 (40.48)		
Yes	44 (51.16)	19 (43.18)	25 (59.52)		
Hypertension, n (%)				-	0.346
No	24 (27.91)	10 (22.73)	14 (33.33)		
Grade I	6 (6.98)	5 (11.36)	1 (2.38)		
Grade II	24 (27.91)	13 (29.55)	11 (26.19)		
Grade III	32 (37.21)	16 (36.36)	16 (38.10)		
Diabetes, n (%)				χ^2^ = 0.41	0.521
No	44 (51.16)	24 (54.55)	20 (47.62)		
Yes	42 (48.84)	20 (45.45)	22 (52.38)		
Renal insufficiency, *n* (%)				χ^2^ = 0.14	0.709
No	65 (75.58)	34 (77.27)	31 (73.81)		
Yes	21 (24.42)	10 (22.73)	11 (26.19)		
Hepatic insufficiency, *n* (%)				χ^2^ = 0.03	0.855
No	50 (58.14)	26 (59.09)	24 (57.14)		
Yes	36 (41.86)	18 (40.91)	18 (42.86)		
PPCI, *n* (%)				χ^2^ = 0.00	1.000
No	82 (95.35)	42 (95.45)	40 (95.24)		
Yes	4 (4.65)	2 (4.55)	2 (4.76)		
AF, *n* (%)				χ^2^ = 0.14	0.708
No	67 (77.91)	35 (79.55)	32 (76.19)		
Yes	19 (22.09)	9 (20.45)	10 (23.81)		
Ventricular aneurysm, *n* (%)				χ^2^ = 0.07	0.796
No	76 (88.37)	38 (86.36)	38 (90.48)		
Yes	10 (11.63)	6 (13.64)	4 (9.52)		
IABP, *n* (%)				χ^2^ = 1.01	0.315
No	16 (18.60)	10 (22.73)	6 (14.29)		
Yes	70 (81.40)	34 (77.27)	36 (85.71)		
Ventilatory support, *n* (%)				χ^2^ = 10.52	0.001
No	50 (58.14)	33 (75.00)	17 (40.48)		
Yes	36 (41.86)	11 (25.00)	25 (59.52)		
Vasoactive drug, *n* (%)				χ^2^ = 11.59	<0.001
No	32 (37.21)	24 (54.55)	8 (19.05)		
Yes	54 (62.79)	20 (45.45)	34 (80.95)		
Infection, *n* (%)				χ^2^ = 9.06	0.003
No	30 (34.88)	22 (50.00)	8 (19.05)		
Yes	56 (65.12)	22 (50.00)	34 (80.95)		
PASP (mmHg), *n* (%)				χ^2^ = 0.97	0.324
≤35 mmHg	57 (66.28)	27 (61.36)	30 (71.43)		
>35 mmHg	29 (33.72)	17 (38.64)	12 (28.57)		
Size, *n* (%)				χ^2^ = 0.50	0.479
≤10 mm	34 (39.53)	19 (43.18)	15 (35.71)		
>10 mm	52 (60.47)	25 (56.82)	27 (64.29)		
Location, *n* (%)				χ^2^ = 0.10	0.756
anterior	58 (67.44)	29 (65.91)	29 (69.05)		
posterior	28 (32.56)	15 (34.09)	13 (30.95)		
LVEDD (mm)	56.00 (49.25, 60.00)	53.50 (49.75, 61.00)	57.00 (49.25, 60.00)	Z = −0.09	0.931
LVEF (%)	47.00 (40.25, 55.75)	48.00 (41.75, 56.00)	45.50 (39.25, 53.75)	Z = −1.12	0.261

Z: Mann-Whitney test, χ^2^: Chi-square test, -: Fisher exact. SBP: systolic blood pressure; HR: heart rate; WBC: white blood cells; CREA: creatinine; CK: creatine kinase; CK-MB: creatine kinase isoenzymes; NT-proBNP: N-terminal pro-brain natriuretic peptide; TNI: troponin I; TG: triglycerides; TC: total cholesterol; HDL: high-density lipoprotein; LDL: low-density lipoprotein; TBIL: total bilirubin; DBIL: direct bilirubin; IBIL: indirect bilirubin; A to D: AMI to Door; CS: cardiac shock; PPCI: primary percutaneous coronary intervention; AF: atrial fibrillation; IABP: Intra-aortic balloon pump; PASP: pulmonary artery systolic pressure; LVEDD: left ventricular end-diastolic dimension; LVEF: left ventricular ejection fraction.

**Table 2:** Results of Logistic regression analysis.

Variables	Univariate Logistic Regression Analysis					Multivariable Logistic Regression Analysis				
	β	SE	Z	*p*	OR (95%CI)	β	SE	Z	*p*	OR (95%CI)
WBC (10^9^/L)	0.17	0.05	3.39	<0.001	1.18 (1.07~1.30)	0.13	0.06	2.05	0.040	1.13 (1.01~1.28)
D-dimer (mg/L)	0.27	0.09	3.01	0.003	1.30 (1.10~1.55)	0.16	0.08	2.02	0.043	1.18 (1.01~1.38)
Early revascularization										
No					1.00 (Reference)					1.00 (Reference)
Yes	−1.73	0.57	−3.05	0.002	0.18 (0.06~0.54)	−1.50	0.70	−2.15	0.032	0.22 (0.06~0.88)
Ventilatory support										
No					1.00 (Reference)					1.00 (Reference)
Yes	1.48	0.47	3.16	0.002	4.41 (1.76~11.06)	1.25	0.60	2.08	0.038	3.48 (1.07~11.29)
Vasoactive drug										
No					1.00 (Reference)					
Yes	0.58	0.51	1.13	0.260	1.78 (0.65~4.88)					
Infection										
No					1.00 (Reference)					1.00 (Reference)
Yes	1.61	0.53	3.01	0.003	5.00 (1.75~14.27)	1.38	0.69	1.99	0.047	3.97 (1.02~15.42)

**Table 3:** Performance comparison of nested models by sequential addition of variables.

Model	Variables	AUC	95% CI	Sensitivity	Specificity	PPV	NPV
Model 1	Early revascularization	0.656	0.567–0.745	0.881	0.432	0.597	0.792
Model 2	Early revascularization + ventilatory support	0.754	0.658–0.850	0.524	0.864	0.786	0.655
Model 3	Early revascularization + ventilatory support + infection	0.767	0.667–0.866	0.762	0.659	0.681	0.744
Model 4	Early revascularization + ventilatory support + infection+ WBC count	0.803	0.744–0.923	0.810	0.795	0.791	0.814
Model 5	Early revascularization + ventilatory support + infection+ WBC count + D-dimer	0.866	0.785–0.946	0.857	0.818	0.818	0.858

PPV: Positive Predictive Value; NPV: Negative Predictive Value.

## Data Availability

The authors agree to make data and materials supporting the results or analyses presented in this paper available upon reasonable request.

## Supplementary Materials

The following supporting information can be downloaded at: https://www.mdpi.com/article/10.3390/jcm15082919/s1, Figure S1: Timeline of variable collection in this study.
