# Supplementary figures and images for: Construction of a Nomogram Prediction Model for Mortality Risk Within 14 Days in Patients with Acute Myocardial Infarction and Ventricular Septal Rupture
Source: J Clin Med. 2026 Apr 11;15(8):2919. doi: 10.3390/jcm15082919 (PMC13115988; doi:10.3390/jcm15082919)

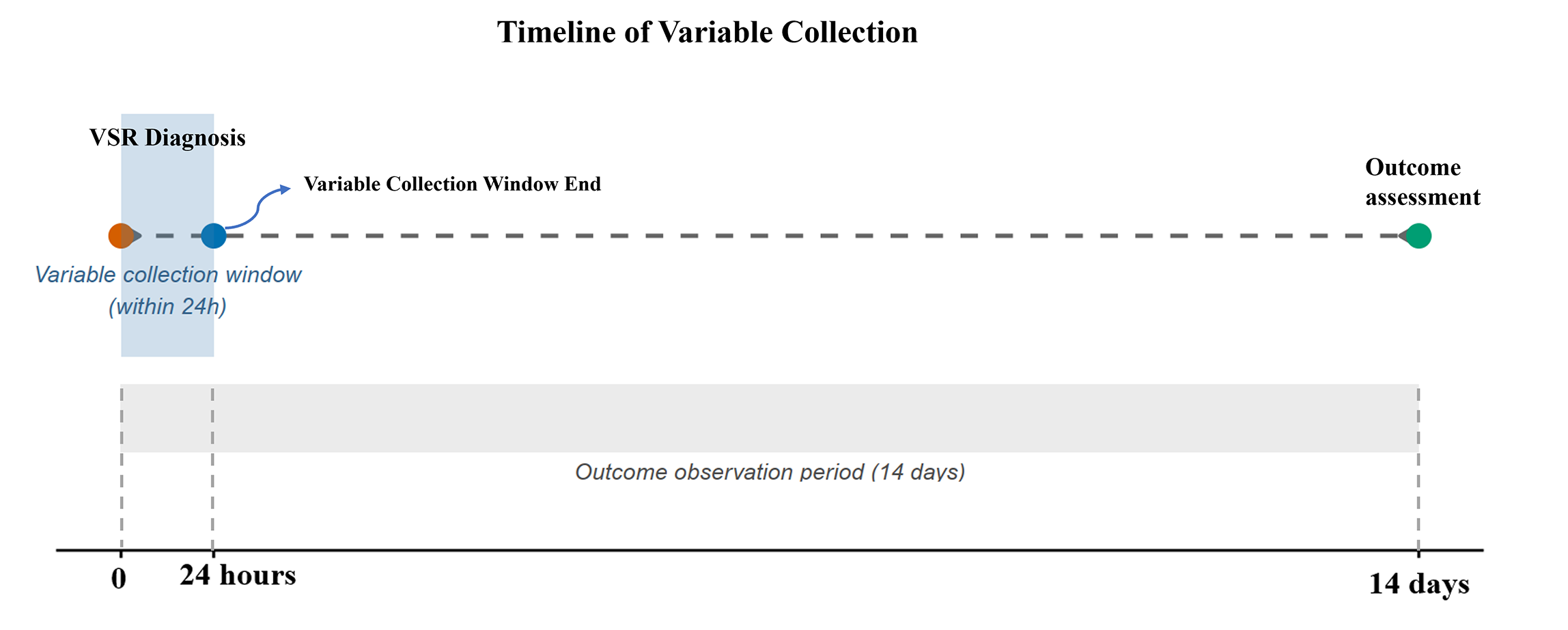

Supplement: Supplementary file 1 [file jcm-15-02919-s001.zip › jcm-4193883-supplementary.tif]
